# Immunological Effects of Aggregation-Induced Emission Materials

**DOI:** 10.3389/fimmu.2020.575816

**Published:** 2020-10-06

**Authors:** Haibo Wu, Wen Huang, Xingyu Zhou, Yuanzeng Min

**Affiliations:** ^1^ Department of Pathology, The First Affiliated Hospital of USTC, Division of Life and Sciences and Medicine, University of Science and Technology of China, Hefei, China; ^2^ Intelligent Pathology Institute, The First Affiliated Hospital of USTC, Division of Life Sciences and Medicine, University of Science and Technology of China, Hefei, China; ^3^ CAS Key Lab of Soft Matter Chemistry, University of Science and Technology of China, Hefei, China; ^4^ Department of Chemistry, University of Science and Technology of China, Hefei, China; ^5^ Department of Endocrinology, The First Affiliated Hospital of USTC, Anhui Provincial Hospital, University of Science and Technology of China, Hefei, China; ^6^ Hefei National Laboratory for Physical Science at the Microscale, University of Science and Technology of China, Hefei, China

**Keywords:** aggregation-induced emission, immunity, inflammation, biomedical imaging, application of aggregation-induced emission

## Abstract

Nanotechnology is widely used in the fields of biology and medicine. Some special nanoparticles with good biocompatibility, hydrophilicity, and photostability can be used as ideal systems for biomedical imaging in early diagnosis and treatment of diseases. Among them, aggregation-induced emission materials are new antiaggregation-caused quenching nano-imaging materials, which have advantages in biocompatibility, imaging contrast, and light stability. Meanwhile, heterogeneity of nanoparticles may cause various adverse immune reactions. In response to the above problems, many researchers have modified nano-materials to be multifunctional nano-composites, aiming at combining diagnosis and treatment with simultaneous imaging and targeted therapy and additionally avoiding immune reactions, which is of great potential in imaging-guided therapy. This review discusses the application of aggregation-induced emission materials, and other nano-imaging materials are also mentioned. We hope to provide new ideas and methods for the imaging of nano-materials in diagnosis and treatment.

## Introduction

Nanotechnology is widely used in the fields of biology and medicine. Among them, nanoparticles (NPs) play a key role in biomedical imaging and the therapeutic fields of modern medicine due to their special physical and chemical properties, such as electrical conductivity, stability, and optical properties. Some NPs, called nano-imaging materials, can be used as imaging agents (with diagnostic capability) in ideal imaging systems. The nano-imaging materials commonly used include carbon nanotubes (CNTs), nano-materials with metal ions, rare earth elements, and a new kind of imaging material named aggregation-induced emission (AIE) materials (1). With the improvement of biomedical imaging technology, nano-imaging materials enable the achievement of early diagnosis and visualization during the treatment process based on their unique optical properties. For example, in order to achieve the efficiency and visualization of the diagnosis and treatment, the multifunctional nano-probes with good photothermal property, high imaging contrast, biological safety, and accurate drug delivery are mainly developed. The multifunctional nano-probes not only realize the various ways of imaging, but also achieve an efficient treatment of neoplastic and non-neoplastic disease by combination therapy (2); nano-imaging material with metal ions can convert absorbed light energy into heat energy, leading to the apoptosis or necrosis of specific lesions, which is often used in the photothermal therapy of tumors at present (3).

**Scheme 1:**
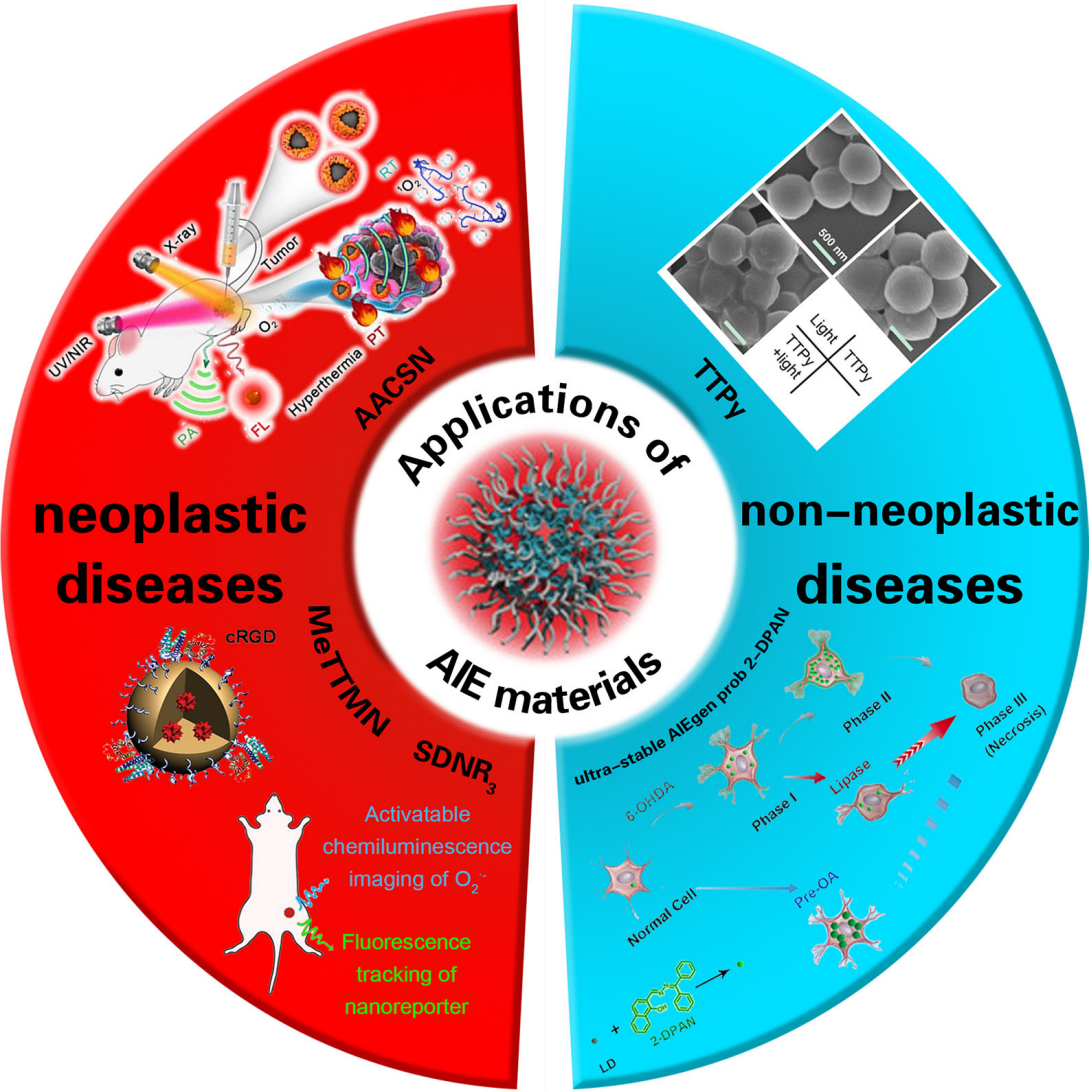
The application of AIE materials in neoplastic and non-neoplastic diseases. cited from Ref (12,) Ref (13), Ref (14), Ref (15) and Ref (16).

In recent years, people have been devoted to the research of a new type of nano-imaging material, namely AIE materials. AIE materials are essential materials about antiaggregation-caused quenching (anti-ACQ), which were first reported in the study by Tang et al. (4) in 2001. It is mentioned in the report that AIE materials can be emitted more efficiently in an aggregated (rather than dispersed) state. In accordance with the advantages of AIE luminogens (AIEgens) in biocompatibility, imaging contrast with biological background, and photostability (5, 6), their potential applications in the fields of fluorescence bioimaging and chemical sensors have attracted widespread attention for research (7) by implementing multimodal imaging, synergistic therapy, and tumor immunotherapy for monitoring as well as effectively generating reactive oxygen species (ROS) as aggregates in order to achieve high-performance fluorescence (FL) imaging-guided photodynamic therapy (PDT) (8). As a result, AIE materials may play an important role in the diagnosis and treatment of neoplastic disease. In addition, AIEgens can be used in the treatment of non-neoplastic diseases. In recent years, antibiotics have been used frequently to cure bacterial infection (9). It is critical to prepare a multifunctional system that has both rapid bacterial differentiation and effective antibacterial properties and to quickly identify gram-positive bacteria in order to achieve an accurate and efficient antibacterial effect (10, 11) (**Scheme 1**).

With the wide application of nano-imaging materials, there are some problems in the safety of these special materials. Some nano-imaging materials can communicate with biological components (like cells, receptors, and proteins) and trigger cell signaling cascades, which can cause an unpredictable immune reaction (activation or suppression) or other negative results (17), such as nondegradability, normal tissue damage, potential immunogenicity, and even cell and systematic immunoreaction and inflammatory reaction (both innate immunity and immunological adaptive reactions) (18). After NPs get into the body, the complement system can be activated by nano-imaging materials, and then they interact with the innate immune system and cause an immunomodulation reaction based on their physical and chemical properties (19). Furthermore, when exposed to the X-ray, an amount of electrons and free radicals damage cell DNA due to the toxic potentially of nano-imaging materials with heavy metal ions. The aqueous solution or physiological solution of the nano-imaging materials used in MRI is less stable and easy to accumulate and precipitate (20), react with plasma proteins, and be phagocytosed by monocytes and macrophages. The damage of some nano-imaging materials, like Ag NPs and Au NPs, to the human body derives from ROS, which break mitochondria function, lysosomes, and cytomembranes and causes apoptosis (21, 22). These imaging materials interact with the cells of the liver, lung, spleen, and skin directly, which will lead to oxidative damage and inflammation (23). Therefore, the key to nanotechnology is to solve heterogeneity when designing nano-materials in order to extend circulation time in the body and achieve long-term immune escape (3). This review emphatically sums up the immunobiological applications related to AIE materials and the solution of potential risks as well as summarizes the recent developments, biological effects, and biomedical applications of other nano-imaging materials.

## The Latest Developments and Biomedical Applications of AIE Materials

NPs with integrated multiple imaging and therapeutic modalities have great potential in accurate diagnosis and improvement of curative effect for tumors. Compared with traditional organic fluorescent materials, AIEgens have higher luminous intensity, photobleaching resistance, and biocompatibility so that they have become a superior tool for biosensing and bioimaging (24, 25).

### AIEgens Can Realize Multimodal Imaging and Synergistic Therapy

Hypoxia in the tumor microenvironment often leads to reduced effectiveness of radiation therapy (RT) for some malignant tumors. However, photothermal therapy (PTT) under near infrared irradiation (NIR) can increase the blood flow and promote the oxygen supply of tumor tissue (26). Resulting from the uneven distribution of heat in tumor tissue, the tumor cannot be eradicated effectively by PTT alone (26). Therefore, combining the uniform irradiation of RT with the oxygen pump effect of PTT is an ideal choice to achieve synergetic treatment of tumors and improve the therapeutic effect. For tumor therapeutics, integrating multiple imaging and treatment modes into a single structural unit in order to attain an accurate diagnosis and improve the therapeutic effect is a promising research interest with profound clinical value (27).

The simple Ag @ AIE core-shell nanoparticles (AACSN) were prepared by Xue et al. (28) using the simple silver core/AIE shell NPs, which realize a new strategy of multimodal imaging and cooperative therapy. The adjustability of shell thickness could help to overcome the incompatibility between FL and plasma noble-metal NP while the excellent performance of FL and CT imaging can also be maintained. More importantly, an additional function is generated on the core-shell interface to achieve outstanding properties of PT and PA. The experimental results show that five types of imaging and therapy modes based on FL, PA, CT, PTT, and RT are successfully constructed in core @ shell nanostructure. This simplifies the complex preparation process and avoids the potential incompatibility between different components. This strategy provides an effective way to design multifunctional nanomaterials for disease diagnosis and synergetic treatment.

### AIEgens Can Improve the Therapeutic Efficiency of PTT and PDT

**Figure 1:**
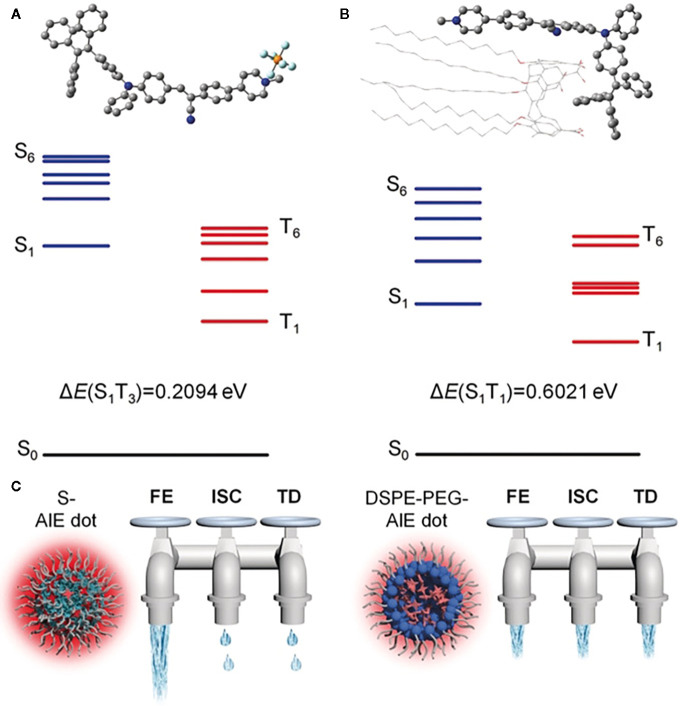
Optimized molecular structures and calculated energy diagrams of **(A)** 1 and **(B)** 1+CC5A-12C complex. **(C)** The three dissipation pathways of the absorbed excitation energy for different AIE dots, which are likened to three water taps. FE, fluorescence emission; TD, thermal deactivation. (Reprinted with permission from Ref (30). Copyright ^©^ 2019 WILEY-VCH Verlag GmbH & Co. KGaA, Weinheim).

For optical materials, photophysical properties play key roles in determining the biomedical function and efficacy of optical agents. When an optical material is in an excited state, it dissipates the energy through three pathways (29): fluorescence emission (FE), intersystem crossing (ISC), and thermal deactivation (TD), which are utilized as FL, luminescence, and PA imaging or PTT, respectively. Regulating the optical agents through a nano-engineering approach or molecular design can enhance one of the three pathways of these agents and improve one of their imaging effects. For the nano-engineering approach, an optical agent can be delivered and aggregated in the tumor and then reacts with the tumor microenvironment and opens up the radiative pathway. For molecular design, one can improve the molecular structure to narrow the energy gap, decrease energy loss, or control the agent’s release, thus enhancing cancer phototheranostics. As is mentioned in previous research (30), one can control the imaging effect of AIEgens by regulating FE, ISC, or TD. Dan Ding et al. use calix arene and AIEgens to form supramolecular AIE dots, which are encapsulated by PEG-12C. These kinds of NPs could restrict ISC and TD, so the light excitation energy will only release by FE (see **Figure 1**), causing highly emissive, photosensitive supramolecular AIE nano-dots.

PDT is an emerging means for tumor treatment. Under the function of a photosensitizer (PS), the cytotoxic ROS are generated, leading to the death of tumor cells (31). This method has the distinct advantages of minimal invasion and high spatiotemporal precision (32, 33). Although preliminary results have been achieved by PDT in the treatment of tumors, there are still the following deficiencies. First, traditional PSs such as rose bengal and methylene blue have the problem of low generation efficiency of ROS, which limits the antitumor activity of PDT (34). Second, the PSs mentioned above exhibit intrinsically weak FL (35) while the lack of FE is not conducive to manipulate FL imaging-guided PDT. Consequently, multifunctional materials need to be designed to improve the therapeutic efficiency of PDT. In recent years, the emergence of PSs with AIE features has promoted the new development of PDT. It is proved that AIEgens can effectively generate ROS as aggregates and then realize high-performance FL imaging-guided PDT (36). Inspired by the advantages of stimuli-responsive nano-micelles and AIE PSs in tumor treatment, the team of You ML and Ben ZT designed two kinds of stimuli-responsive nano-micelles carrying a far red–emissive AIE PS (MeTTMN) to improve the generation efficiency of ROS and the PDT effect (37). Two kinds of stimuli-responsive polymer, mPEG-Hyd-PCL-CIN (P-Hyd) and mPEG-SS-PCL-CIN (P-SS), were successfully synthesized (8). The results show that the synthesized polymer has good biocompatibility, spontaneous assembly into nano-micelles in aqueous solution, and good drug-loading ability (AIE-PS-MeTTMN with high drug loading). In addition, compared to the control group, the generation efficiency of ROS can be significantly improved by using stimulus-responsive nano-micelle carriers at a simulative cancer environment. These MeTTMN-loaded stimuli-responsive nano-micelles have an efficient inducing effect on the apoptosis of tumor cells. Jun D and Ben ZT et al. (38) efficiently made fluorogen TTB with AIE properties encapsulated within a polymeric matrix and modified with RGD-4R peptide to prepare RGD-4R-MPD/TTB NPs with NIR emission, high photostability, and low dark cell toxicity. The results show that the PDT based on RGD-4R-MPD/TTB NPs T can effectively inhibit the growth of cervical, prostatic, and ovarian cancer. By observing changes of tumor histology and protein levels, it was found that it could effectively promote the apoptosis and necrosis of tumor cells, inhibit the proliferation of tumor cells, and thereby promote the death of cells. These results suggest that the efficiency of FL imaging-guided PDT could be improved in different ways, and the NIR PS with AIE character might be used as a substitute for nano-probes and nano-medicines in the clinical treatment of various tumors.

### The Monitoring of AIEgens in Tumor Immunotherapy

**Figure 2:**
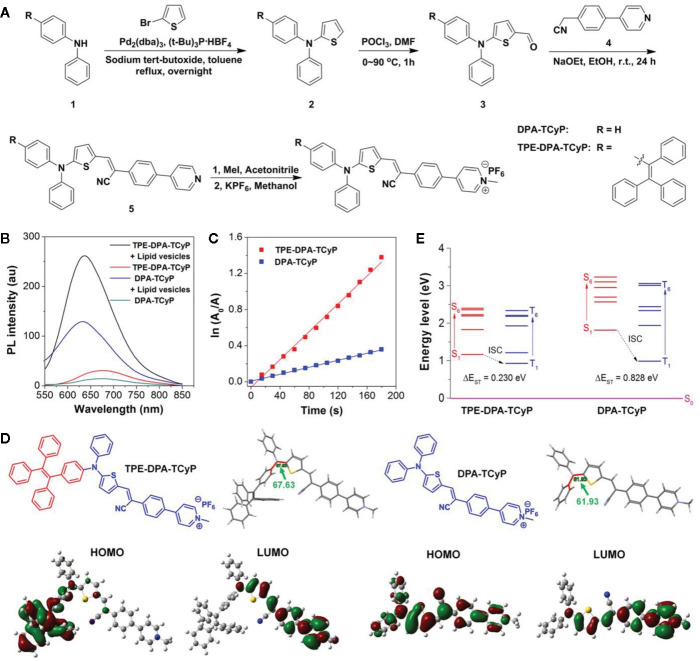
**(A)** Synthetic route to TPE-DPA-TCyP and DPA-TCyP. **(B)** Photoluminescence (PL) spectra of TPE-DPA-TCyP and DPA-TCyP (10×10^-6^_M_) in the presence and absence of lipid vesicles (22×10^-6^_M_) in PBS. **(C)** Plot of ln(A_0_/A) against light exposure time, where A_0_ and A are the ABDA absorbance (378 nm) before and after irradiation, respectively. **(D)** Chemical structures, dihedral angles, and HOMO-LUMO distributions by DFT calculations of TPE-DPA-TCyP and DPA-TcyP. **(E)** Energy levels of S_1_-S_6_ and T_1_-T_6_ calculated by the vertical excitation of the optimized structures in **(D)**. (Reprinted with permission from (12) Copyright ^©^ 2020 Wiley-VCH Verlag GmbH & Co. KGaA, Weinheim).

As a new type of luminescent material, AIEgens has become a powerful tool for biological sensing and monitoring, including long-term tracking (39). The tumor immunotherapy provides new options for the treatment of various types of malignant tumors. It aims to train the immune cells in the host so as to destroy the tumor cells, but the response in patients is generally limited (40). Hence, the immune reaction needs to be monitored *in vivo* in volunteers to optimize the immunotherapeutic effect. At present, the existing methods include the determination of whole blood lymphocytes and immunocytokine and biopsy of tumor tissue. However, these measures are invasive and cannot effectively reflect the data of dynamic therapy (41). ROS plays a key role in regulating biological functions from intracellular homeostasis to cell death. What is more, it is essential for ROS to activate immune reactions (42). In innate immunity, the phagocytes (such as neutrophils and macrophages) can spontaneously promote the generation of ROS and fight against infection through an oxidation mechanism (17). In an adaptive immune reaction, the activation of T cell receptors triggers the generation of ROS in T cells, leading to the activation of T cells and cytokine secretion (19). In conclusion, the ROS can be used as a biomarker to monitor immune activation. Dan Ding et al. (12) carried out a novel PS, which reduced intermolecular interaction with a twisted donor-π-acceptor (D-π-A) molecular structure, which could also restrict the excited-state intramolecular motion due to the steric hindrance (see **Figure 2**). With consumption of absorbed excitation energy decreasing, more ROS were induced, and immunogenic cell death was massively evoked. In recent years, molecular imaging technology has wide prospective application in real-time evaluation of immunoactivation *in vivo* in volunteers. Although fluorescent probes can be used to detect ROS (13), they are rarely used to detect ROS in immune cells because of their inadequate biodistribution and poor sensitivity, let alone for *in vivo* imaging of immune activation. D. Cui and K. Pu et al. (14) synthesized a kind of semiconducting polymer nano-reporters (SPNRs) with superoxide anion (O_2_•^−^), which can activate chemiluminescence signals for *in vivo* imaging of immunoactivation during tumor immunotherapy. Among them, SPNR3 represents the first O_2_•^−^-activatable near-infrared chemiluminescent reporter. Owing to its high selectivity and sensitivity, the SPNR3 can distinguish the higher O_2_•^−^ levels in immune cells from that in other cells (including tumor and normal cells). After systemic administration, SPNR3 preferentially accumulates in the tumor cells of living mice and activates the chemiluminescence signal in the tumor microenvironment. In addition, the improvement of *in vivo* chemiluminescence signals after tumor immunotherapy is related to the increase of T cells in tumors, which indicate the feasibility of SPNR3 tracking T cell activation. Therefore, this kind of AIEgens can be used for real-time imaging of tumor immunotherapy *in vivo*, and it has great prospective application for high-throughput screening of immunotherapeutics of immunotherapy drugs.

In recent years, much research has been done on the biomedical applications of AIEgens involved immunological effects. For example, Wang et al. (43) discovered an FL self-reporting approach based on AIE properties to monitor polymeric fluorescent particles (PFPs). PFPs with uniform and tunable sizes harboring the abilities of biolabeling and photosensitizing, can be employed as superior optical nano-agents for photo-controllable immunotherapy. NK cells keep their original immunocompetence after coating with low-concentration PFPs, and such immunocompetence could be promoted by PFPs under light irradiation in PFP-coated NK cells. In addition, PFP-coated NK cells promote immunotherapy efficiency to cancer cells. Thus, excellent optical nano-agents greatly boost the extensive applications of precipitation polymerization in various science and technology areas.

### The Role of AIEgens in the Treatment of Non-Neoplastic Diseases

#### AIEgens for the Diagnosis and Treatment of Bacterial Infectious Diseases

Human health is seriously threatened by bacterial infectious diseases, especially those caused by gram-positive bacteria (44). Millions of people are infected with gram-positive bacteria every year (45); furthermore, such bacteria causes 25% of surgical-site infections in nosocomial infections (46). In addition, in some kinds of tumors, the gram-positive bacteria can significantly enhance the resistance of chemotherapy drugs, reduce the curative effect of chemotherapy drugs (2), and promote tumor growth and metastasis (47). The traditional methods of bacterial identification include a gram-staining test, plate-culture, polymerase chain reaction, and immunological methods (48), but the implementation of them requires complex instruments, much time, large amounts of labor, and expensive fees (49). In recent years, antibiotics have been used frequently to cure bacterial infection (9). Although they are capable of killing bacteria and are easily accessible, excessive use and abuse of antibiotics inhibit their effectiveness and develop drug resistance (50). Therefore, in the case of no drug resistance, it is critical to prepare a multifunctional system that has both rapid bacterial differentiation and effective antibacterial properties and to quickly identify gram-positive bacteria in order to achieve an accurate and efficient antibacterial effect (10, 11). FL imaging-guided photodynamic antibacterial technology serves as an effective method to solve this problem in recent years (15). Michelle M.S. Lee et al. (15, 51) use AIE-active molecules called TTVP with good water solubility, NIR emission, and extremely high generation efficiency of ROS for the first time to carry out bacterial identification and photodynamic antibacterial research. The research indicates that TTVP can selectively target gram-positive bacteria through a washing-free and ultrafast staining procedure after the incubation period of 3 s, which shows ultrafast bacterial identification. The results of *in vitro* and *in vivo* experiments show that TTVP can completely inactivate gram-positive bacteria under white light irradiation *in vitro*, so it is a kind of super strong light-mediated antibacterial. More significantly, it also has a significant effect on photodynamic antibacterial treatment in a rat model of skin wound infection. This was the first time it has been reported that NIR-emissive AIEgen as a multifunctional agent can effectively kill gram-positive bacteria *in vivo* and *in vitro* with both specific identification and photodynamics, which provide guidance for the rational design of easy-to-operate and time-saving bacterial identification reagents and the promotion of the development of high-performance antibacterial materials.

#### The Diagnosis and Treatment of Parkinson’s Disease (PD) With AIEgens

PD is one of the most common progressive neurodegenerative diseases, which often occurs in people aged 60 years and over with symptoms of shaking palsy and involuntary tremble (52). However, the exact cause of the disease is unknown (53). Recently, studies have shown that lipid droplets (LDs) serve as containers of triglycerides and cholesteryl esters as well as the dynamic organelles for lipid metabolism, protein storage, signal regulation, and cell apoptosis (54). It was found by Liu et al. (55) that mitochondrial disorder and oxidative stress lead to accumulation of LDs, and the oxidized lipid metabolites further promote mitochondrial disorder and cause neuronal death and PD, which indicates LDs may play an important role in PD’s progress. Thus, the real-time monitoring of LDs from PD patients is of great value. Li HL et al. (16) synthesized ultra-stable AIEgen probe 2-DPAN to monitor the dynamic process of LDs in PD model cells. The results show that LDs are closely related to the change of mitochondrial activity; that is, lipase can accelerate the process of cell death, prestimulate LDs through unsaturated fat acid oleic acid (OA), and reduce the process of cell death by inhibiting the production of excess ROS and fat acid so as to protect the mitochondria. Therefore, real-time behavior monitoring of LDs is important and necessary in the early stage of PD prevention. The application of 2-DPAN proves the importance of LDs in neuronal homeostasis, and the effective regulation of LDs may prevent or inhibit the progress of PD. Benefiting from its good specificity, photostability, and biocompatibility, the probe may become a useful tool for studying LD-related diseases.

## The Disadvantage and Improvement of AIE Materials and Nano-Imaging Materials

### Deficiencies and Modification of AIEgens

Based on the research on AIEgens in the past 20 years, it can be seen that the introduction of AIE can solve the problem of fluorescent quenching. However, there are still some limitations, such as high hydrophobicity, poor cell compatibility, short emission wavelength, low penetration, long latency (> 5 min), and being time-consuming (56, 57), which severely limit the biological applications of AIE materials *in vivo*. To develop and promote the biomedical applications of fluorescent organic NPs based on AIE materials, their biocompatibility and the application of cell imaging are further studied. The hydrophobic surface of NPs can be modified to be hydrophilic so that they can have good water dispersibility and novel AIE properties. Xi QZ et al. (58) have designed a new system of FL bioprobes based on nano-aggregates combining AIE-based organic fluorogens An18 (derived from 9,10-distyrylanthracene with an alkoxyl end group) and surfactant Pluronic F127. It was also the first time to propose a simple method for preparing AIE-based fluorescent organic nanoparticles (FONs) by mixing AIE units (An18) and surfactant F127. Originating from the hydrophobic interaction between An18 and F127, water-soluble An18-F127 NPs are widely used in cell imaging. The research shows that the modified AIE-based NPs are biocompatible with cells and easy to observe. It means that the surfactant-modified AIE-based FONs have good water solubility, biocompatibility, and a convenient preparation method, which can be used as a new kind of bioimaging dye.

### Deficiencies and Modification of Nano-Imaging Materials

**Figure 3:**
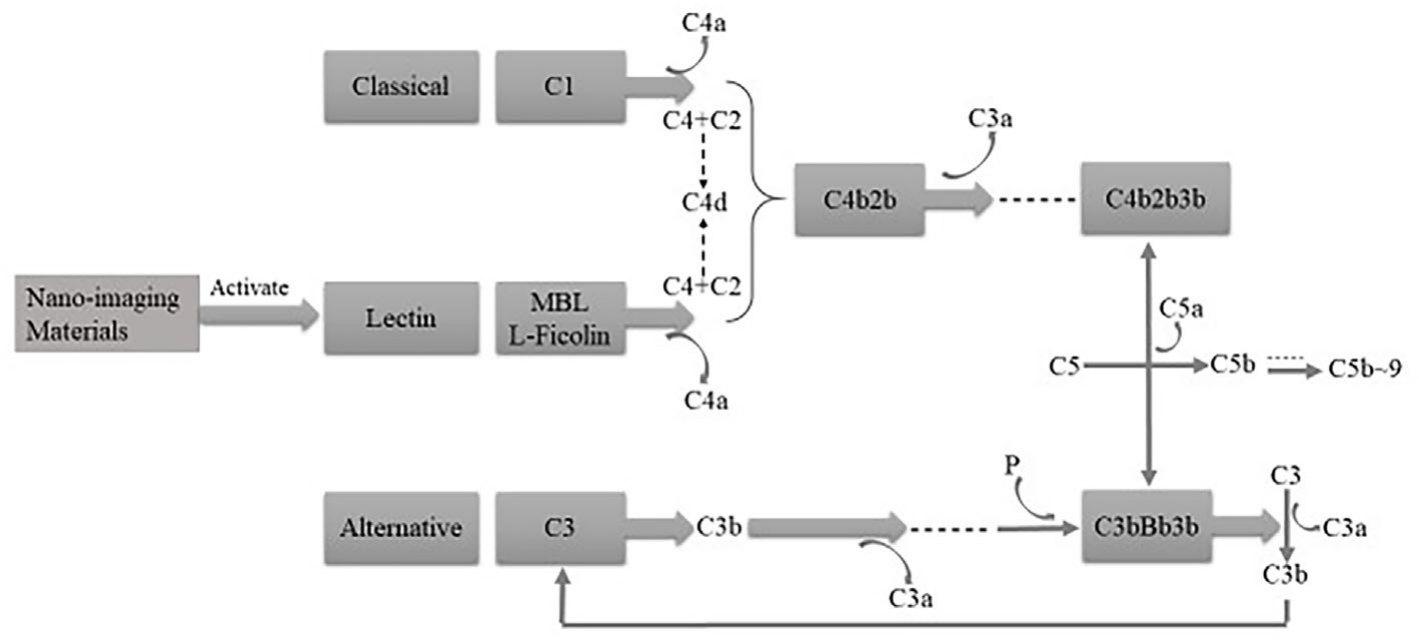
The complement system can be activated by three pathways, that is, classical, alternative, and MBL pathways.

As an important part of innate immunity, the complement system is the first defense to deal with invaders and protect the body, and it can be activated by three pathways, i.e., the classical, alternative, and mannan-binding lectin (MBL) pathways. After NPs get into the body, they interact with the innate immune system and generate the immunomodulation reaction based on their physical and chemical properties (19). With the participation of the complement members (see **Figure 3**), the acute anaphylactoid reaction or anaphylactic reaction would be triggered. In order to reduce the adverse reactions and toxicity, it is feasible to use surface modification technology and restrain the formation of complement C3 and C5 invertase so as to improve innate immune compatibility and safety *in vivo*.

Because of high X-ray absorbance, metal nano-imaging materials can be used as radio sensitizers. Nevertheless, this is potentially toxic because of the heavy metal ions. The aqueous or physiological solution of the magnetic NPs (MNPs) used in MRI is less stable and easy to accumulate and precipitate (20), resulting in the decrease of effective concentration in the tissue. Thus, ligand choosing is the premise and basis for optimizing and stabilizing the optical properties of a nanocluster and reducing its toxicity. In addition, according to the physical and chemical properties of NPs, the multifunctional NPs of biocompatibility would be manufactured after the modification (59) for the immune escape and *in vivo* longevity.

The damage of some nano-imaging materials, like Ag NPs and Au NPs, to the human body derives from ROS, which could interact with the cells of the liver, lung, spleen, and skin directly, leading to oxidative damage and inflammation (23). Cytoskeleton damage, mitochondrial activity, and the changes of protein and nucleus metabolism are the main effects of NPs on the cells. Studies of cytotoxicity and genotoxicity show that toxicity of some nano-imaging materials to tumor cells led to cytoskeleton damage and impact cell division, which explain the potential applications and mechanism of nano-imaging materials on malignant tumor therapy.

## Conclusion

With the rapid development of the emerging field of nanomedicine, nano-imaging materials are applied to daily life and medicine. Functional/smart AIEgen probes have made great progress in specific bacterial imaging and killing, targeted cell/intracellular organelle imaging and ablation, and targeted tumor therapy. At present, biomedical imaging is mainly used for the diagnosis and therapy for image capture. Previous studies demonstrate that most of AIEgens harbor low *in vitro* cytotoxicity, and one of the limitations of AIEgens is that they are not good at building FL “turn-on” nanoprobes. Therefore, complex molecular design and modification must be carried out to obtain multi-functional nano-imaging materials (60). Great efforts have been made to modify new AIEgens and traditional nano-imaging materials for the decrease of the immune reaction and to synthesize multifunctional nano-composite particles through the design of nanostructures, which provides a new method for realizing image-guided tumor therapy and multimode imaging and collaborative therapy. Researchers have designed various multifunctional nano-composite particles based on photothermal conversion and the improvement of mediating tumor oxygen levels, which are beneficial for the therapy of combination photothermal and radiation. Additionally, nano-imaging materials can enhance the targeting of drug delivery as the chemotherapy drug carrier and make tumor targeting imaging and combined therapy (chemotherapy and radiotherapy) possible by combining PTT and MRI diagnosis. Similarly, the synthesis and modification of AIEgens through different ways could improve the efficiency of FL imaging-guided PDT, NIR PS with AIE character could be the substitutes of nano-probes and nanomedicines for multiple tumor clinical therapies and real-time supervision and imaging during tumor immune therapy, optimizing the effect of the immune therapy. Nano-imaging materials can not only be used in malignant tumor therapy, but also in benign diseases as probes, which has a high prospective application in diagnosis and treatment.

Nano-imaging materials should be given more attention and research in the future due to their high medical and biomedical value as well as the new ideas and possibilities for people in the field of disease therapy.

## Author Contributions

HW, WH, and XZ contributed equally. All authors contributed to the article and approved the submitted version.

## Conflict of Interest

The authors declare that the research was conducted in the absence of any commercial or financial relationships that could be construed as a potential conflict of interest.
